# Implications of root morphology and anatomy for water deficit tolerance and recovery of grapevine rootstocks

**DOI:** 10.3389/fpls.2025.1541523

**Published:** 2025-03-20

**Authors:** David Alonso-Forn, Ignacio Buesa, Luis Flor, Antoni Sabater, Hipólito Medrano, José M. Escalona

**Affiliations:** ^1^ Agro-environmental and Water Economy Research Institute, University of Balearic Islands (INAGEA-UIB), Palma, Spain; ^2^ Research Group of Plant Biology Under Mediterranean Conditions, University of Balearic Islands (PlantMed-UIB), Palma, Spain; ^3^ Dept. of Ecology and Global Change, Desertification Research Center (CIDE; CSIC-UV-GVA), Valencia, Spain

**Keywords:** climate change, drought, hydraulic conductivity, plant water status, root biomass, root length density, *Vitis* spp., xylem diameter

## Abstract

The intensification of drought conditions due to climate change poses a major challenge to sustainable grape production. Rootstocks are essential in supporting grapevine water uptake and drought resilience; however, their physiological responses to water stress are not fully understood. Under the hypothesis that root morphology and anatomy may be key traits in grapevine tolerance to water deficit, this study aimed to investigate these traits across diverse rootstocks under progressive water deficit and recovery phases. Thirteen genotypes, including commercial rootstocks and recently bred RG-series and RM2, were evaluated over two seasons in controlled pot-based conditions. Plants were subjected to five distinct watering stages, from well-watered to severe drought. Root traits, such as length, density, and xylem anatomical features, were analyzed alongside stem water potential (Ψ_stem_) to gauge plant water status. Results showed significant genotype-specific differences in root morphology and anatomy, impacting drought tolerance and recovery. Rootstocks with higher root length density (RLD) and a larger proportion of fine roots maintained Ψ_stem_ more effectively under severe drought. Additionally, smaller xylem vessel diameters and reduced xylem area relative to root cross-sectional area correlated with improved water transport efficiency and faster recovery post-drought. A trade-off emerged wherein increased root density enhanced water uptake capacity but came at the cost of reduced transport efficiency. Notably, rootstocks 420A, 41B, RM2, and Fercal displayed superior drought resilience, while the RG-series did not outperform established genotypes like 13-5 Evex, 110 Richter, and 140 Ruggeri. These results underscore the role of root morphology and anatomy in grapevine drought tolerance, suggesting that these traits could be incorporated as criteria for future rootstocks breeding programs. Nevertheless, field-testing under non-limiting soil conditions is essential to validate these findings.

## Highlights

- A higher proportion of fine roots and smaller xylem vessel diameters appear to be key traits in conferring a better tolerance to severe water deficit and in enhancing recovery capacity.
- A trade-off between root architecture and anatomy on stem water potential is particularly noteworthy.
- The 420A presents root morphological and 41B had anatomical features that could give them advantages against severe water deficit and increase their recovery capacity.
- Fercal and the new breed rootstock RM2 possess both, root morphological and anatomical characteristics, that seems to provide them greater resistance to severe water deficit and enhanced recovery capacity.
- The RG series rootstocks do not demonstrate clear advantages in terms of stress adaptation compared to commercial rootstocks.

## 1 Introduction

Over recent decades, climate change has significantly impacted global viticulture due to increasingly warmer and drier conditions (van Leeuwen and Destrac-Irvine, 2017; IPCC, 2023). Rising temperatures, altered precipitation patterns and extreme weather events have negatively impacted grape production and quality (Fraga et al., 2016; Dayer et al., 2017). In the Mediterranean region, drought is expected to exacerbate in frequency and duration increasing soil water deficit and jeopardizing the sustainability of viticulture (Moriondo et al., 2013; Costa et al., 2016). Adapting Mediterranean viticulture to climate change may entail the selection of grapevine genotypes better adapted to drought (Costa et al., 2012; Medrano et al., 2015; Romero et al., 2018; Tortosa et al., 2016; 2019). Each genotype (cultivars, clones and rootstocks) possesses intrinsic characteristics that facilitate this adaptation (Bota et al., 2016; Dayer et al., 2020a; Romero-Azorín and García-García, 2020; Buesa et al., 2022).

Grapevine rootstocks —*Vitis* genotypes used as a base for grafting specific grape cultivars (*Vitis vinifera* L.)— are selected for their rooting characteristics suited to the local environment, as well as pests and diseases resistance and adaptability, which in turn influences vine health and productivity (Ollat et al., 2015; Marín et al., 2021). Their role in tolerating biotic and abiotic stressors, including drought, is well documented (Gambetta et al., 2012; Serra et al., 2014; Lavoie-Lamoureux et al., 2017; Romero et al., 2018; Buesa et al., 2023). Nevertheless, rootstocks have a narrow genetic base (Riaz et al., 2019), with only 10 rootstocks are used for grafting for about 90% of grapevine cultivars worldwide (Serra et al., 2014). Thus, new rootstocks are being developed to address evolving pests and diseases, and future climate conditions (Ollat et al., 2015; Merli et al., 2016; Bordenave et al., 2014). An example is the ‘RG-series’ and RM2 rootstocks, which could confer drought stress tolerance and can differentially control vine vigor and yield (Ramsing et al., 2021; Marín et al., 2021; Buesa et al., 2023).

Despite the significance of rootstocks in drought stress physiology, mechanisms underlying its adaptations are poorly understood due to its multi-trait nature (Gambetta et al., 2012; 2020; Marín et al., 2021; Reyes, 2021), especially regarding hydraulic traits (Tramontini et al., 2013). Vines exhibit high hydraulic conductivity due to their long and wide vessels, although their degree of xylem vulnerability remains controversial (Braun and Schmid, 1999; Choat et al., 2010; Hochberg et al., 2016; Venturas et al., 2016). This seems to be related to the fact that vines have a bimodal vessels distribution typical of climbers, with very wide but also narrow xylem vessels (Jacobsen et al., 2015; Haj-Yahya et al., 2024). Numerous hydraulic traits determine grapevine response to soil water deficit, for example; xylem anatomy (Gambetta et al., 2012; Hochberg et al., 2015; Santarosa et al., 2016; Dayer et al., 2017), aquaporin regulation (Gambetta et al., 2012; 2013; Pou et al., 2013), root suberization (Barrios-Masias et al., 2015; Cuneo et al., 2021), hormonal dynamics (Rogiers et al., 2012; Dayer et al., 2020b), osmotic adjustment (Martorell et al., 2015; Sorek et al., 2021), root morphology (Alsina et al., 2011; Peccoux et al., 2017), stomatal regulation (Medrano et al., 2002; Merli et al., 2016), hydraulic conductance adjustment (Romero et al., 2010; Buesa et al., 2023), and vessels embolism vulnerability (Venturas et al., 2016; Lamarque et al., 2023) and its ability to repair (Zufferey et al., 2011; Knipfer et al., 2015). All these traits are related to the hydraulic traits of cultivars, rootstocks and their interaction (Serra et al., 2014; Peccoux et al., 2017; Gambetta et al., 2020). Therefore, a first approach to understand the above-mentioned traits is to start from the roots (Marín et al., 2021).

The roots and the vascular system play a pivotal role in soil water uptake and transport under soil water deficit conditions (Gambetta et al., 2012; Zhang et al., 2016; Marín et al., 2021). Previous studies have highlighted root morphological and anatomical parameters as key to drought tolerance and water use efficiency (de Herralde et al., 2006; Alsina et al., 2007, 2011; Cuneo et al., 2021). Several authors have attributed genotype differences in the response to water deficit to variations in the morpho-anatomy of specific tissues (Pratt, 1974; Hochberg et al., 2015; Dayer et al., 2017; Zhu et al., 2017; Reingwirtz et al., 2021; Lamarque et al., 2023; Pérez-Álvarez et al., 2023). In other grafted woody species, such as *Prunus* spp (Tombesi et al., 2010), *Malus* spp (Bauerle et al., 2011), *Olea* spp (Trifilò et al., 2007). and *Populus* spp (Fichot et al., 2009). a positive correlation between xylem vessel diameter of rootstocks and maximal hydraulic conductivity was observed. However, in rubber (*Hevea brasiliensis*), wood xylem vessel density, and not vessel diameter, was related to hydraulic efficiency (Waite et al., 2023). In addition, it has been reported some degree of phenotypic plasticity in morphological traits in response to evaporative demand rather than soil moisture, likely due to xylem acclimation (von Arx et al., 2012; Brunner et al., 2015). In grapevines, root segments of ‘Shiraz’ showed a reduction in xylem vessel size under water deficit (Mapfumo et al., 1994). In petioles, xylem differentiation also occurs in response to water deficit in both ‘Shiraz’ and ‘Cabernet Sauvignon’ grapevines (Hochberg et al., 2015), as well as in ‘Chasselas’ (Dayer et al., 2017). In grapevine leaves and stems, Palliotti et al. (2011) reported that the smaller mean xylem vessel diameter of ‘Sangiovese’ than ‘Montepulciano´s’ was related to the lower hydraulic conductance of the former, suggesting that it would probably be less susceptible to conduit damage. These changes, commonly observed in woody plants (Brodribb, 2009; Álvarez and Sánchez-Blanco, 2013; Fonti et al., 2013), lead to reduced hydraulic conductivity. Similarly, Pouzoulet et al. (2020) found the higher xylem vessel density in ‘Thompson Seedless’ stems compared to ‘Merlot’, relating to higher hydraulic conductivity. A trade-off between hydraulic efficiency and vulnerability has been hypothesized (Schultz, 2003; Lens et al., 2011; Pouzoulet et al., 2014; Quintana-Pulido et al., 2018). However, this trade-off has not been confirmed across aboveground (branches and trunks) and belowground (roots) organs in tree species (Lübbe et al., 2022).

Despite the root’s importance in whole plant water relations, they have received much less attention than aboveground parts due to soil accessibility limitations (Branas and Vergnes, 1957; Marín et al., 2021). Therefore, additional research is required to understand how root system vascular traits affect plant hydraulics under different soil water conditions. In this sense, scarce literature is available regarding response under severe water stress and recovery of grapevine rootstocks (Reyes, 2021). This study aims to identify morphological and vascular root traits related to plant water status regulation under different soil water availabilities by evaluating several grapevine ungrafted rootstocks, both commercial and novel RG-series and RM2. For this purpose, an evaluation conducted under potted conditions through gradual water deficit and subsequent recovery seems appropriate (Blackman et al., 2019; Ruehr et al., 2019).

## 2 Materials and methods

### 2.1 Plant material and experimental conditions

**Figure 1:**
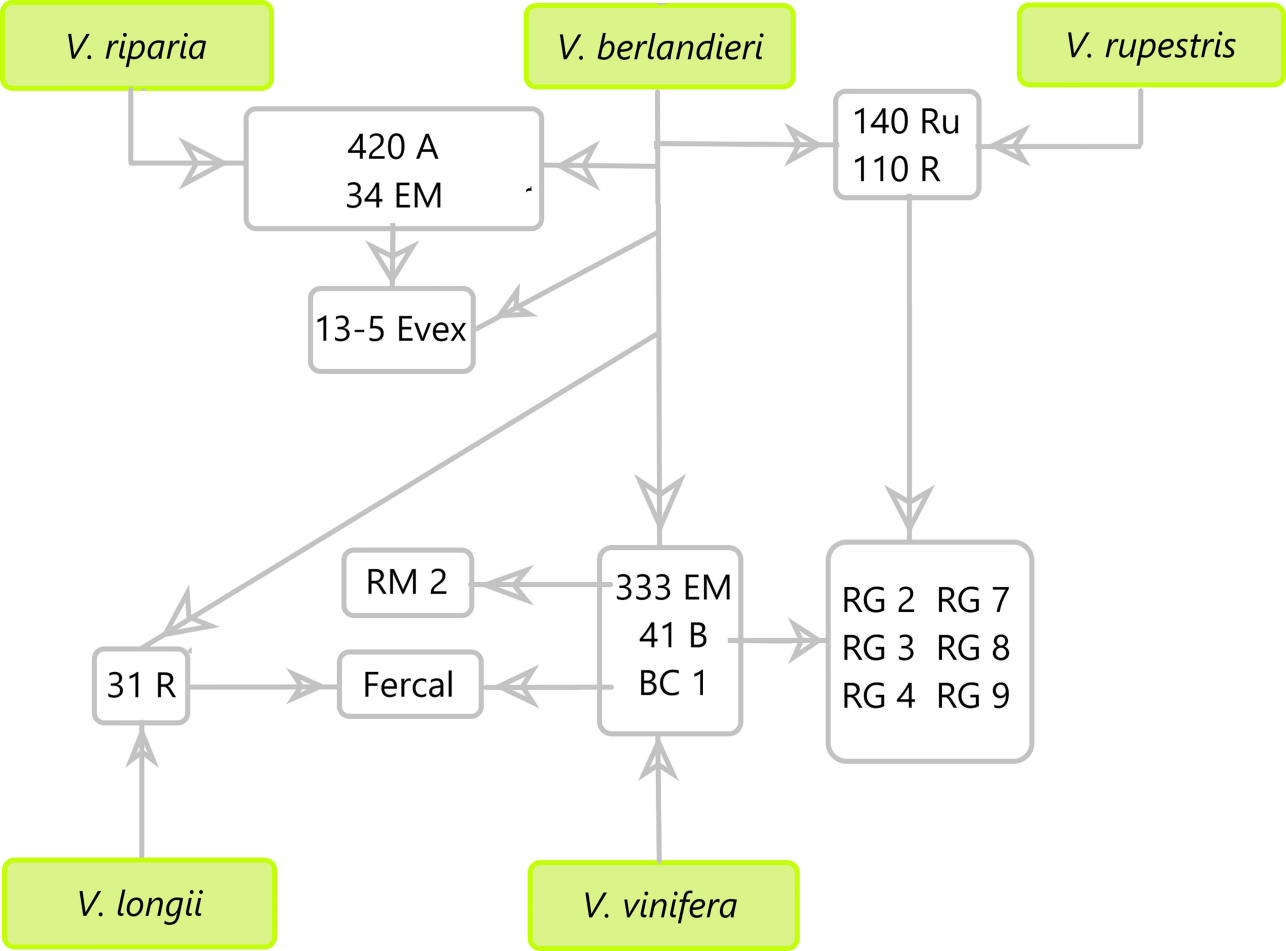
Parental scheme of the 13 rootstocks tested in this study (420A, 13-5 Evex, 140Ru, 110R, RM2, Fercal, 41B, RG2, RG3, RG4, RG7, RG8, and RG9).

The experiment was conducted at the experimental field of the University of Balearic Islands, Spain (39°38’14.8”N, 2°38’51.4”E). The plants, which were two years old, were placed in 23-liter pots after having their roots trimmed to 5 cm in length to ensure that the evaluated root developed during the experiment. Six plants per genotype were grown outdoor and fertigated to ensure no nutritional deficiencies. Seven commercial and six new breeding rootstocks were used covering a great genetic diversity of parents of the *Vitis* genus (420A MGt (420A), Millardet et Grasset 41B (41B), 13-5 Evex, Fercal, 140 Ruggeri (140Ru), 110 Richter (110R), RG2, RG3, RG4, RG7, RG8, RG9 and RM2). Within these rootstocks it can be found combinations of *V. riparia* × *V. berlandieri:* 420 *A*; of Chasselas Blanc (*V. vinifera)* × *V. berlandieri*: 41B; 34 EM (*V. riparia* × *V. berlandieri*) × *V. berlandieri*: 13-5 Evex; of 31 R (*V. longii* × *V. berlandieri*) × BC 1 (*V. vinifera* × *V. berlandieri*): Fercal; of *Vitis berlandieri* × *V. rupestris* parents: 140Ru and 110R; of 110R × 41B: RG-series; and of *V. vinifera* × 333 EM (*V. berlandieri*): RM2 (**Figure 1**).

**Figure 2:**
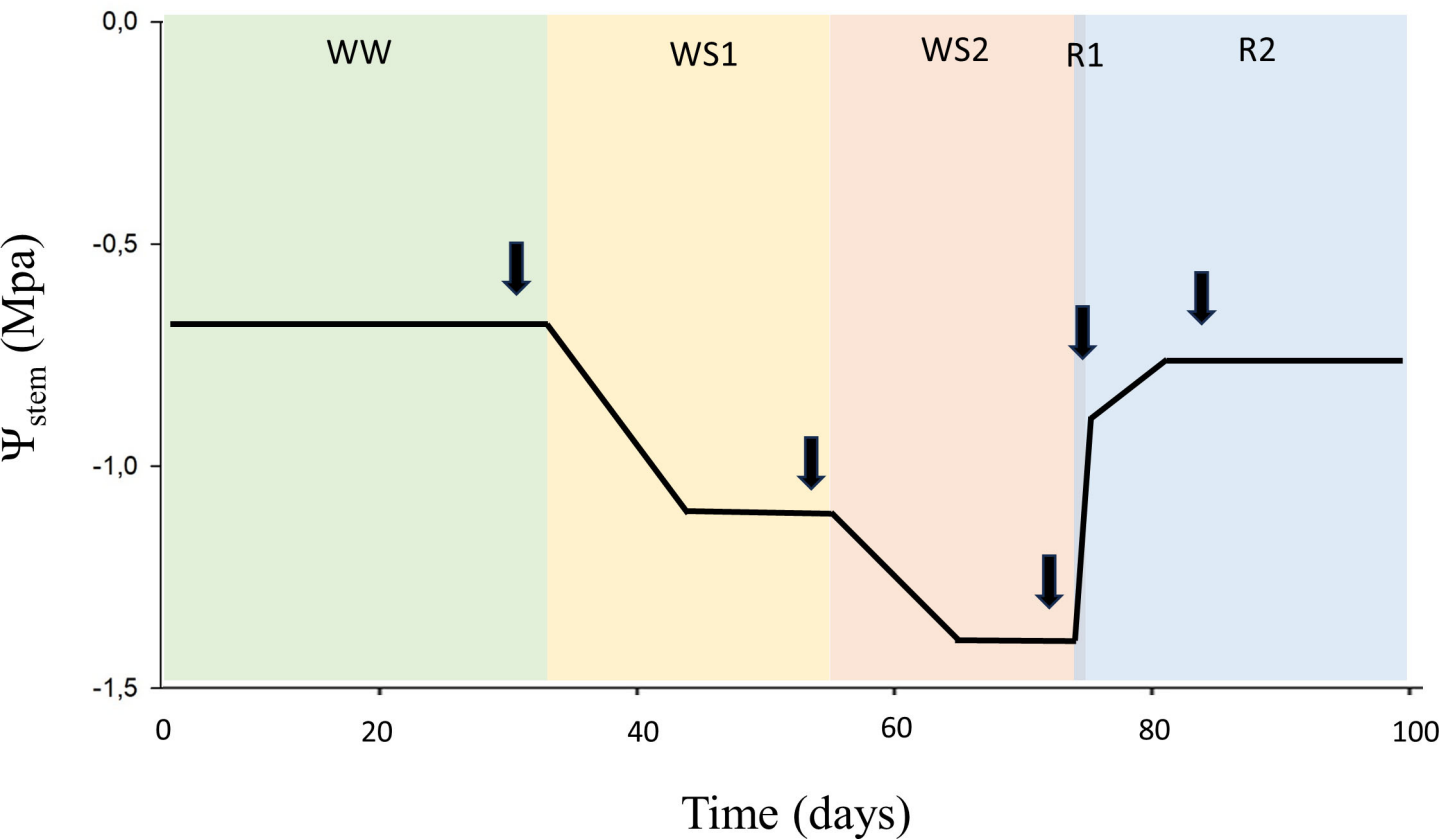
The figure illustrates the five hydraulic conditions experienced by the rootstocks throughout the experiment. Arrows indicate the timing of measurements; black line represents the overall mean values of stem water potential. The X-axis corresponds to the accumulated days of the experiment, the Y-axis to stem water potential. Each colour on the background represents an experimental condition: well-watered (WW); moderate water deficit (WS1); severe water deficit (WS2); short-term recovery (R1) and long-term recovery (R2).

To evaluate the response of the different rootstocks to water deficit and recovery, a two-year study was conducted during 2021 and 2022 seasons. The rootstocks studied in 2021 were 420A, 41B, RG2, RG4, RG8, and RM2; while those studied in 2022 were 140Ru, 110R, RG3, RG7, RG9, Fercal, and 13-5 Evex. In both years, the plants were initially maintained at field capacity until reaching a height of 1.5 meters. A progressive water deficit was then imposed over two months, followed by a 10-day recovery period during which irrigation was restored to field capacity. The water stress level of the plants was determined by measuring early morning stomatal conductance (g_s_) rates by means of an infrared open gas exchange analyzer system (Li-6400xt, Li-cor Inc.). This resulted in five soil water conditions in which the rootstocks were evaluated: well-watered (WW), moderate water deficit (WS1), severe water deficit (WS2), short-term recovery (R1), and long-term recovery (R2) (see **Figure 2**). Each vine was irrigated twice per day through two pressure-compensated drippers of 0.5 L h^-1^. The irrigation applied was between 1 and 1.5 L/day during the WW period, thereafter, irrigation was stopped for 6 days, followed by a progressive sustained deficit irrigation of 0.6 L/day over 22 and 26 days in 2021 and 2022, respectively. Afterwards, a quick water recovery was applied by hosepipe irrigation, followed by drip irrigation of 1.5 L/day.

### 2.2 Water relations

Measurements were conducted on non-stressed plants (WW), under two levels of drought stress (WS1 and WS2), after 24 hours of recovery (R1) and after 10 days of recovery (R2). Stem water potential (Ψ_stem_) was measured using a pressure chamber (Model 600, PMS Instruments, USA) at solar noon (12:30-13:30 solar time) on one fully expanded leaf per plant (n = 6), previously covered for 1 hour in darkness inside a zip-lock bag with a metallized high-density polyethylene reflective film (Sonoco RF, Sonoco Products Co., Hartsville, South Carolina, USA) (Choné et al., 2001).

### 2.3 Root morphology

Prior to measuring root morphological and anatomical parameters, the roots were carefully extracted from the pot of each plant at the end of each year of the experiment (n = 6). They were then cut at the root crown, and the substrate was washed off. Morphological parameters of the whole root system were captured by using an EPSON Expression 10000 XL scanner (Epson America, Inc., Long Beach, CA, USA). Subsequently, the obtained images were analyzed utilizing the WinRHIZO software (Regent Instruction, Quebec, Canada), following the protocol outlined in Lupo et al. (2022). From this image analysis, a series of root morphological parameters were calculated: total length (m), average root diameter (mm). Specific root area was calculated by dividing the root area by the root dry biomass, as detailed in Lõhmus et al. (1989). Additionally, root length density (RLD), defined as the root length (cm) per volume of soil (cm^3^), was calculated according to Lupo et al. (2022) and grouped into 3 categories: less than 0.5 cm cm^-3^, between 0.5 and 1 cm cm^-3^, and greater than 1 cm cm^-3^. Roots were oven-dried at 65°C for 72 hours and root dry biomass was determined.

### 2.4 Root anatomy

#### 2.4.1 Principio del formulario

Anatomical parameters of the xylem vascular system in the main roots of all plants were meticulously analyzed (n = 6) (Mapfumo et al., 1994). At 8-10 cm from the root tip, 4-6 cm segments were sampled. These sections were immersed in a solution consisting of formaldehyde, acetic acid, and ethanol (70%) in a ratio of 0.5:0.5:9 for a period of 48 hours. Tissue segments were then subjected to dehydration using a graded ethanol series (50%, 70%, 95%, and 100%, each for 30 minutes), followed by immersion in tert-butanol for 8 hours and embedding in paraffin wax (Paraplast Plus, Leica, Wetzlar, Germany).

Subsequently, cross-sections meticulously prepared using a microtome (RM2235, Leica, Nussloch, Germany) and affixed to glass slides. The samples underwent a process of de-paraffinization, rehydration, and staining with aniline blue (Lupo et al., 2022). The images acquired with the optical microscope were meticulously analyzed using the Image J software (version 2.9; Schindelin et al., 2012) to derive the desired parameters (**Supplementary Figure 1**). These parameters encompassed the xylem vessel density (V_D_, number of vessels per mm^2^), xylem diameter (D, in μm) measured as equivalent circle diameter of xylem area, and percentage of xylem area in relation to the total area were calculated based on Santarosa et al. (2016).

Following Hagen-Poiseuille equation (Tyree and Ewers, 1991), the theoretical specific hydraulic conductivity (*k_th_*, kg s^−1^ m^−1^ MPa^−1^) was calculated as follows:


$$k_{th}=\sum (\left(\pi *D^{4}*\rho \right)/\left(128*\eta \right))*1/A_{xyl}$$

Where *D* represents the equivalent circle diameter (m), *ρ* stands for the density of water (at 20°C, 998.2 kg m^-3^), *η* denotes the viscosity of water (at 20°C, 1.002 × 10^-9^ MPa s), and *A_xyl_* represents the xylem area (m^2^).

### 2.5 Statistics

The data underwent a normality distribution analysis using the Shapiro-Wilk test, analysis of variance ANOVA, and mean comparisons were conducted using the Duncan test with a significance level of 95% (p-value< 0.05). In addition, correlation analysis was conducted using the Pearson method. The entire statistical analysis was carried out using R Studio software (R Core Team, 2023). R: A Language and Environment for Statistical Computing. R Foundation for Statistical Computing, Vienna, Austria).

## 3 Results

### 3.1 Stem water potential

**Figure 3:**
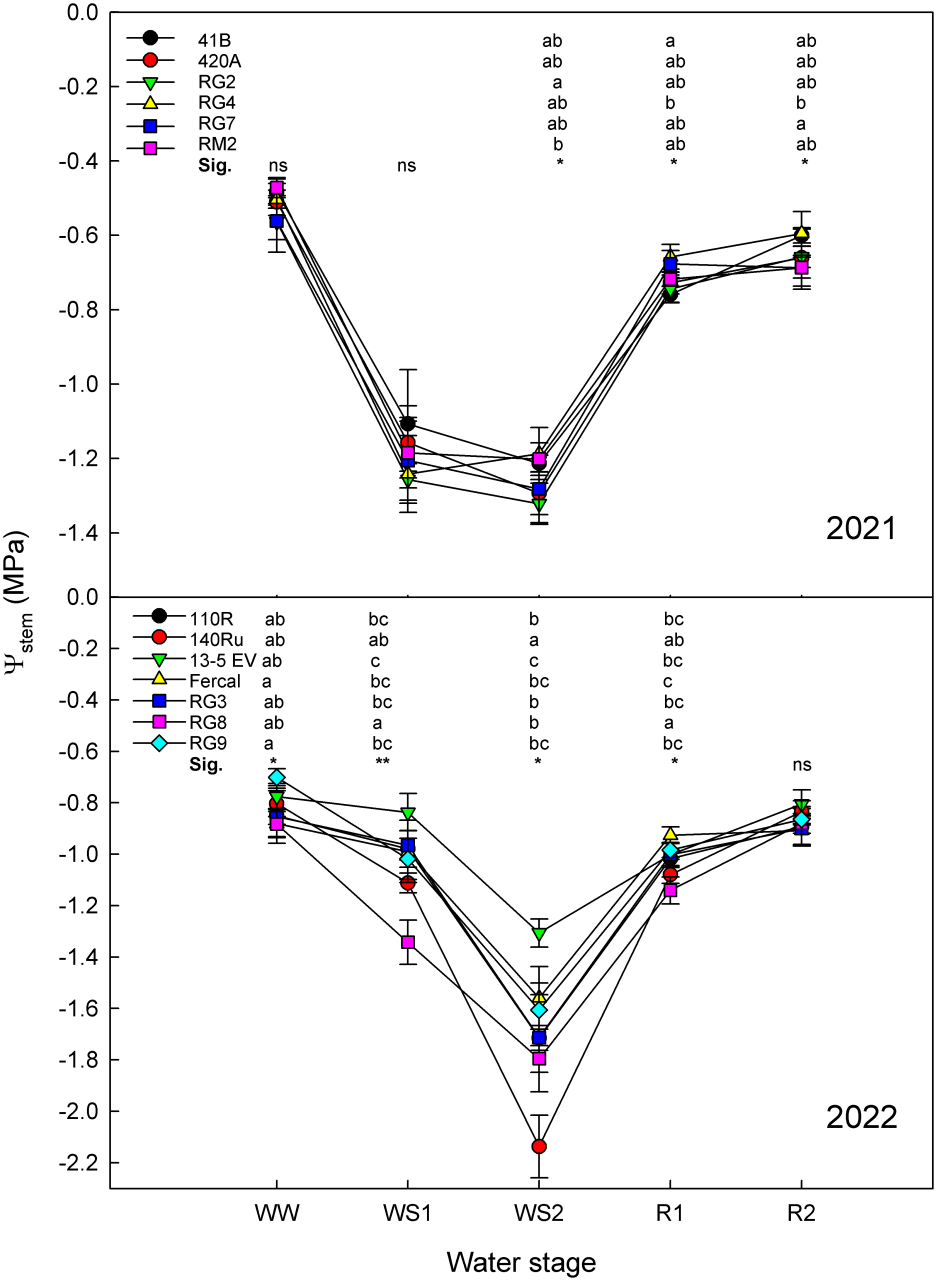
Stem water potential of all the rootstock genotypes studied in 2021 and 2022 at the five water stages: well-watered (WW), moderate water stress (WS1), severe water stress (WS2), short-term recovery (R1), and long-term recovery (R2). Error bars denote standard error (n = 6). Within each water stage, different letters indicate significant differences among genotypes. * and ** mean p-value of <0.05 and <0.01, respectively. ns, non-significant.

Plant water status, assessed by means of Ψ_stem_ showed significant differences between genotypes within each season (**Figure 3**). In both seasons, the Ψ_stem_ transitioned from having the least negative mean values under WW conditions, to obtaining the most negative values under WS2. After the R2, but not R1, the Ψ_stem_ recovered to values comparable to those observed initially under WW conditions.

During 2021, the Ψ_stem_ ranged from -0.47 MPa in WW conditions to -1.32 MPa in WS2 (**Figure 3A**). Significant differences among genotypes were only observed under WS2, R1 and R2 water stages, showing a non-fully consistent trend across all genotypes. Under severe water deficit conditions (WS2), the RG2 showed the most negative Ψ_stem_ values while RM2 the least. During the recovery stages, the RG4 stood out for displaying the least negative Ψ_stem_ while the 41BG and RG7 the lowest.

During 2022, the Ψ_stem_ Values ranged from -0.70 MPa in WW to -2.13 MPa in WS2 (**Figure 3B**). Significant differences among rootstocks were observed at WW, WS1, WS2 and R1. Under WW, WS1, and R1 conditions, the RG8 showed the most negative Ψ_stem_, whereas under WS2, the most negative was reached by140Ru. On the contrary, 13-5 Evex showed the least negative Ψ_stem_ values at WS1 and WS2 and Fercal the fastest recovery at R1.

### 3.2 Root morphology

Root morphological traits showed a wide range of variation between experimental seasons for the studied genotypes (**Table 1**). For instance, total root length ranged from 448 to 1533 m in 2021, and from 191 to 750 m in 2022. Similarly, root dry biomass ranged from 66 to 150 g in 2021, and from 22 to 71 g in 2022. Mean values for specific root area (SRA) ranged from 18.2 to 25.0 m^2^ kg^-1^ in 2021, and from 23.3to 40.2 m^2^ kg^-1^ in 2022. As for root length density (RLD), these varied between 2.0 to 6.7 cm cm^-3^ in 2021, and 0.8 and 3.3 cm cm^-3^ in 2022. The root diameter (ø) (values for the studied genotypes in 2021 ranged from 0.76 to 0.96 mm, and in 2022, from 0.90 to 1.35 mm.

**Table 1:** Root morphology traits of the 13 rootstock genotypes in (A) 2021 and (B) 2022.

Year	Genotype	Total root length (m)	Root dry mass (g)	Specific Root Area (SRA, m_2_ kg_-1_)	Root Length Density (RLD, cm cm^-3^)	Root diameter (mm)
2021	41B	858 ± 83 bc	92.7 ± 3.2 bc	23.7 ± 1.5	3.7 ± 0.4 bc	0.82 ± 0.03 b
	420A	1533 ± 215 a	150 ± 10 a	22.7 ± 2.01	6.7 ± 0.9 a	0.76 ± 0.02 b
	RG2	574 ± 78 cd	68.6 ± 1.4 d	24.3 ± 2.7	2.5 ± 0.3 cb	0.96 ± 0.05 a
	RG4	662 ± 79 cd	77.0 ± 7.4 cd	24.7 ± 1.0	2.9 ± 0.3 cb	0.94 ± 0.03 a
	RG7	448 ± 53 d	65.8 ± 5.4 d	18.2 ± 1.4	2.0 ± 0.2 d	0.87 ± 0.06 ab
	RM2	1034 ± 149 b	103 ± 11 b	25.0 ± 2.8	4.5 ± 0.7 b	0.79 ± 0.04 b
	Significance	***	***	0.254	***	***
	Mean	878 ± 67	94.8 ± 4.7	23.3 ± 0.8	3.8 ± 0.3	0.85 ± 0.02
2022	140Ru	366 ± 44 bc	56.8 ± 1.8 b	23.3 ± 1.6 c	1.6 ± 0.19 bc	1.24 ± 0.1 a
	Fercal	750 ± 83 a	71.3 ± 4.2 a	28.1 ± 1.6 bc	3.3 ± 0.36 a	0.90 ± 0.05 b
	110R	191 ± 24 d	21.9 ± 3.1 e	34.3 ± 3.6 ab	0.8 ± 0.1 d	1.18 ± 0.08 a
	RG9	221 ± 43 cd	29.8 ± 4.4 de	29.7 ± 2.0 bc	1.0 ± 0.18 cb	1.35 ± 0.06 a
	RG8	446 ± 80 b	47.0 ± 5.8 bc	29.3 ± 3.6 bc	1.9 ± 0.35 b	0.97 ± 0.04 b
	RG3	338 ± 54 bcd	39.3 ± 7.3 cd	28.8 ± 1.8 bc	1.5 ± 0.23 bcd	0.98 ± 0.02 b
	13-5 Evex	439 ± 61 b	33.9 ± 4.0 de	40.2 ± 2.5 a	1.9 ± 0.27 b	0.98 ± 0.03 b
	Significance	***	***	***	***	***
	Mean	397 ± 29	43.2 ± 2.4	30.5 ± 1.1	1.7 ± 0.1	1.1 ± 0.03

Total length; root dry mass, specific root area (SRA), root length density (RLD) and mean diameter. Mean values are mean ± standard error (n = 6). Different letters indicate statistically significant differences (P< 0.05) between genotypes. Mean values for each trait are also shown.

*** means p-value <0.001.

In both years, significant differences among genotypes were found in all morphological traits (**Table 1**). In 2021, the genotype 420A exhibited the highest mean values for root length, root dry biomass, RLD, and SRA, but the lowest mean value in root diameter. Conversely, genotype RG7 showed the opposite trend in root length, root dry biomass, RLD, and SRA. The highest diameter was experienced by genotype RG2 and RG4. Increasing the average root diameter was a consistent trend in all RG-genotypes. In 2022, Fercal genotype displayed the highest mean values for root length, root dry biomass, RLD, and SRA, but the lowest root diameter. On the contrary, genotype 110R showed the lowest root length, root dry biomass, and RLD. The highest diameter was observed in genotype 140Ru, 110R and RG9.

**Figure 4:**
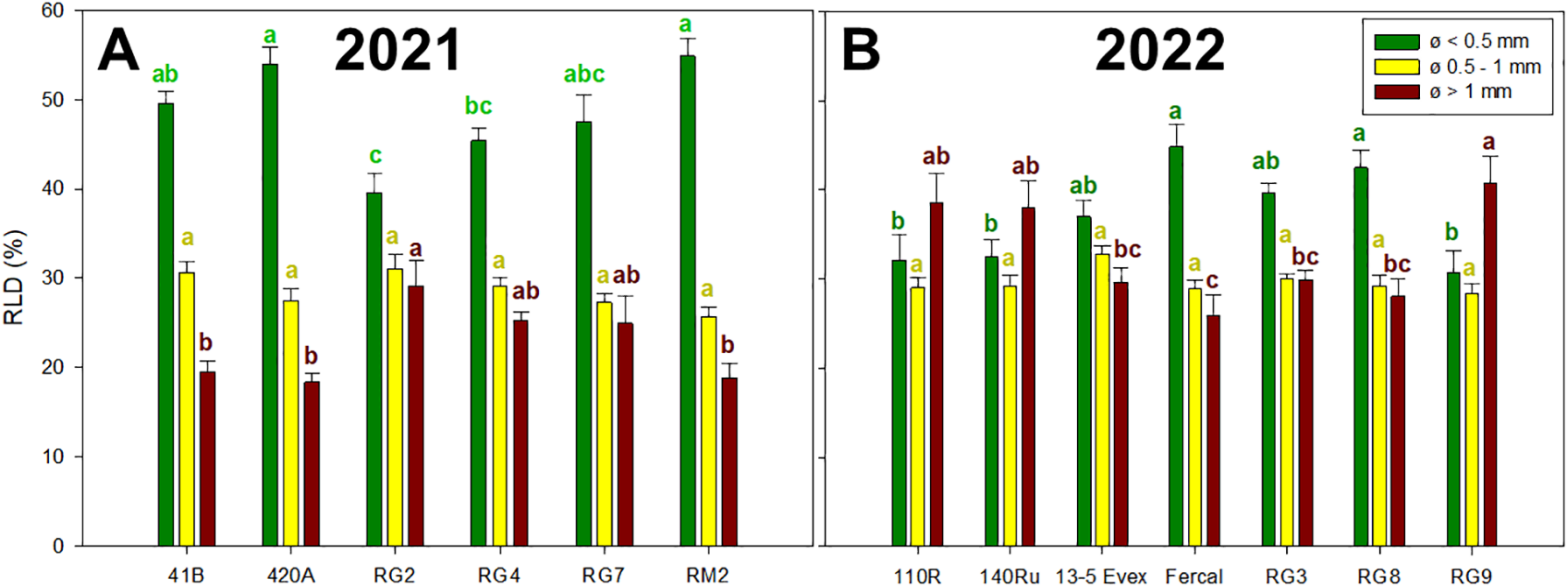
Mean values of root length density per class (RLD, %) are represented in the Y-axis: root length density generated by fine roots (ø< 0.5 mm), intermediate root sizes (ø = 0.5-1 mm) and thick roots (ø > 1 mm). Rootstock genotypes studied for each year are presented on the X-axis: **(A)** for rootstocks studied in 2021, and **(B)** for rootstocks studied in 2022. Different lowecase letters denote significant differences (p-value < 0.05) within each chart colour.

In both years (**Figure 4**), significant variations in the percentage of RLD generated by different root diameters were identified among genotypes and within classes. In 2021, the proportion of total RLD generated by fine roots (ø< 0.5 mm), intermediate root sizes (ø = 0.5-1 mm) and thick roots (ø > 1 mm) ranged from 39 to 54%, 25 to 31%, and from 18 to 29%, respectively (**Figure 4A**). In 2022 (**Figure 4B**), these values ranged from 30 to 44%, from 28 to 32%, and from 25 to 40% for the root classes of fine, intermediate and thick, respectively. In 2021, the RG genotypes tended to have a lower proportion of thick roots and a higher proportion of fine roots compared to 41B, 420A and RM2. In 2022, the 110R, 140Ru and RG9 had lower proportion of thick roots than Fercal and RG8 and the opposite trend was observed for the fine roots.

### 3.3 Root anatomy

Root anatomical traits also showed a wide range of variation between genotypes and years (**Table 2**). For instance, the xylem diameter spanned from 24.9 and 32.0, and from 42.9 and 55.1 µm in 2021 and 2022, respectively. Xylem vessel density (V_D_) ranged from 30 to 41, and from 27.7 to 49.8 vessels mm^-2^ in 2021 and 2022, respectively. The theoretical specific hydraulic conductivity (*k*_th_) varied between 29.4 to 74.8, and from 122.8 and 206.2 kg s^-1^ m^-1^ MPa^-1^ in 2021 and 2022, respectively. The average xylem area relative to the total section area (A_xyl_-to-A_tot_) varied between 2.1 and 3.7, and between 5.7 and 9.4% in 2021 and 2022, respectively.

**Table 2:** Vascular anatomy traits of the 13 studied rootstock genotypes in (A) 2021 and (B) 2022.

Year	Genotype	Xylem diameter (µm)	Xylem vessel density (V_D_, n vessels per mm^2^)	Theoretical specific hydraulic conductivity (*k*_th_, kg s^-1^ m^-1^ Mpa^-1^)	A_xyl_/A_total_ (%)
2021	41B	24.9 ± 1.4 c	41.0 ± 5.3	29.4 ± 3.8 c	2.1 ± 0.2 c
	420A	32.0 ± 1.8 a	37.4 ± 1.9	74.8 ± 6.9 a	3.7 ± 0.3 a
	RG2	30.6 ± 2.5 ab	37.8 ± 5.5	61.6 ± 10.4 ab	3.1 ± 0.2 ab
	RG4	27.3 ± 1.1 bc	31.7 ± 3.4	45.1 ± 5.9 bc	2.1 ± 0.1 c
	RG7	30.3 ± 2.9 abc	30.0 ± 2.7	73.9 ± 17.4 a	2.6 ± 0.3 bc
	RM2	25.4 ± 0.9 bc	39.2 ± 4.2	43.0 ± 7.5 bc	3.4 ± 0.4 bc
	Significance	*	0.353	***	***
	Mean	28.6 ± 0.8	36.5 ± 1.6	55.1 ± 4.1	2.7 ± 0.2
2022	140Ru	55.1 ± 6.3	27.7 ± 4.9 c	206.2 ± 43.1	8.7 ± 1.4 ab
	Fercal	47.9 ± 2.0	27.9 ± 2.4 c	122.7 ± 11.4	5.7 ± 0.5 d
	110R	48.2 ± 4.1	42.5 ± 3.9 ab	139.3 ± 22.5	8.8 ± 0.6 ab
	RG9	52.0 ± 2.1	30.2 ± 2.5 c	154.9 ± 14.7	7.4 ± 0.7 bcd
	RG8	49.1 ± 3.4	44.6 ± 3.2 a	138.5 ± 22.6	9.4 ± 0.9 a
	RG3	52.1 ± 4.2	31 ± 4.6 bc	129.3 ± 16.5	6.3 ± 0.5 cb
	Evex 13-5	42.9 ± 2.4	49.8 ± 6.1 a	123.7 ± 13.9	8.2 ± 0.6 abc
	Significance	0.295	***	0.137	***
	Mean	49.5 ± 1.3	35.6 ± 1.8	142 ± 7.73	7.6 ± 0.3

Mean xylem diameter (D); xylem vessel density (V_D_), vessels per mm^2^ (n); theoretical specific hydraulic conductivity (*k_th_*), and xylem area/total area A_xyl/_A_total_. Values are mean ± standard error (n = 6). Different letters indicate statistically significant differences (P< 0.05) between genotypes. Mean values for each trait are also shown.

* and *** mean p-value of <0.05 and <0.001, respectively.

In both years, root anatomy showed significant differences among genotypes in most of the studied traits (**Table 2**). In 2021, the 420A exhibited the highest values of xylem diameter, V_D_, *k*_th_, and A_xyl_-to-A_tot_ ratio (**Table 2A**). In this season, significant differences among genotypes on V_D_ were not found. Conversely, genotype 41B showed the opposite trend, showing the lowest values for all three traits. In 2022, the genotypes RG8 and 13-5 Evex exhibited the highest xylem V_D_, whereas 140Ru, Fercal and RG9 showed the lowest (**Table 2B**). Regarding the A_xyl_-to-A_tot_ ratio, RG8 the highest mean values whereas Fercal displayed the lowest. In this season, no significant effect of the genotype on xylem diameter and *k*_th_ was detected.

**Figure 5:**
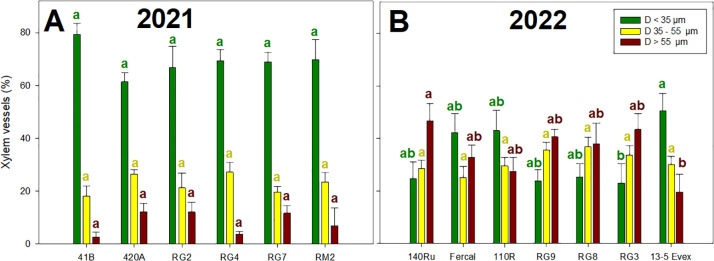
Mean percentage of xylem vessels per class are represented in the Y-axis: xylem diameter thinner than 35 µm (D< 35 µm), xylem diameter between 35-55 µm (D 35-55 µm), and xylem diameter greater than 55 µm (D > 55 µm). Rootstock genotypes studied for each year are presented on the X-axis: **(A)** for rootstocks studied in 2021, and **(B)** for rootstocks studied in 2022. Different lowecase letters denote significant differences (p-value < 0.05) within each chart colour.

In both years (**Figure 4**), significant variations in the percentage of xylem vessels sizes were identified among genotypes and within size (**Figure 5**). In 2021, the proportion of fine xylem vessels (D< 35 µm), intermediate xylem vessels (D 35-55 µm), and thick xylem vessels (D > 55 µm) ranged from 55 to 84%, 14 to 28%, and from 0 to 16%, respectively (**Figure 5A**). In 2022 (**Figure 5B**), these values ranged from 18 to 45%, 25 to 38%, and from 26 to 50%. In 2021, no effect of genotype on xylem vessels size distribution was detected, while in 2022 differences were observed in the percentage of high and low diameters. Specifically, the RG3 had significantly lower proportion of big xylem vessels than 13-5 Evex, while 140Ru displayed higher proportion of small ones than 13-5 Evex.

### 3.4 Regression analysis

**Figure 6:**
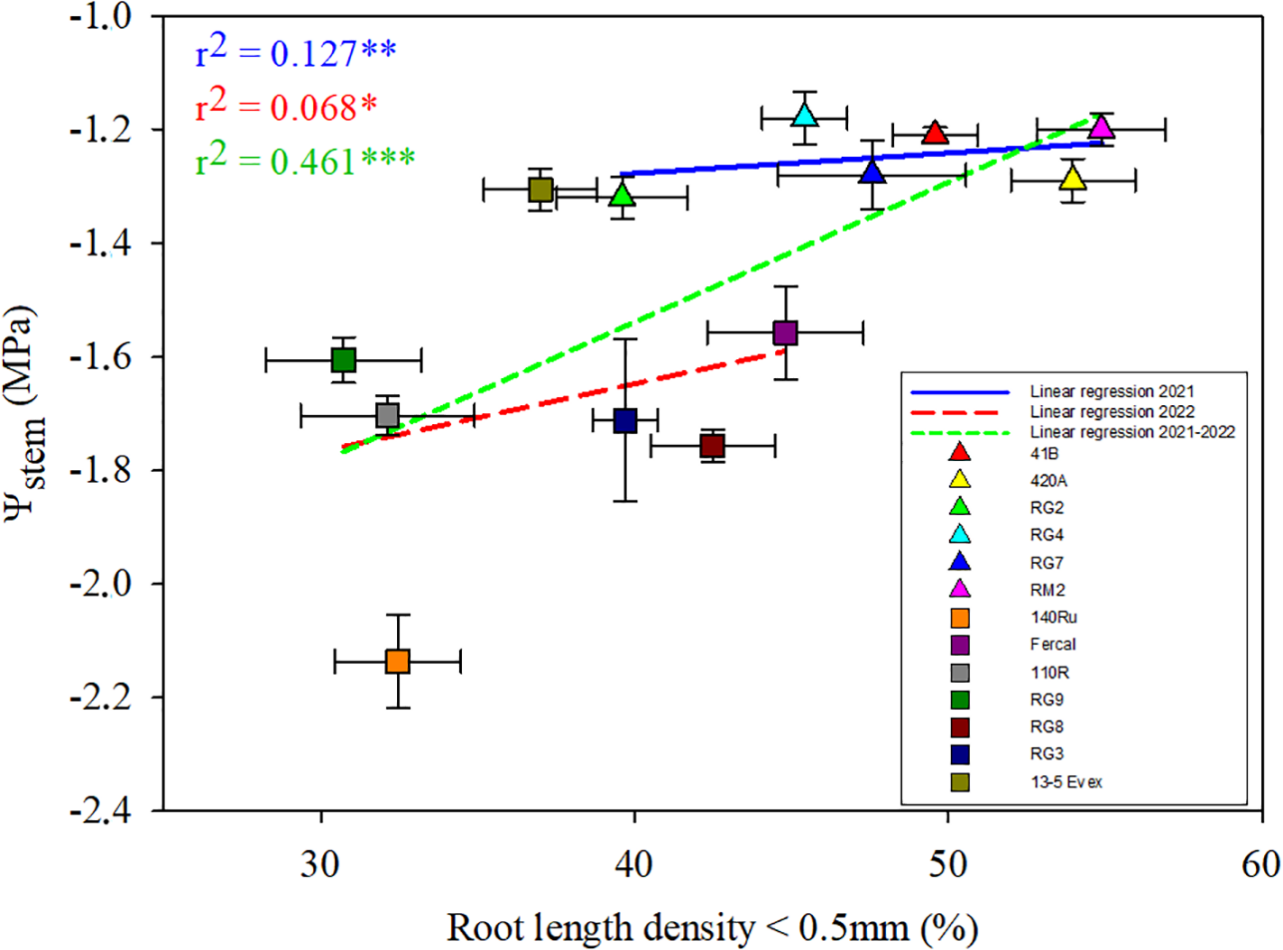
The figure illustrates a multiple linear regression analysis of the percentage of root length density, specifically considering roots with a diameter thinner than 0.5 mm, against Ψ_stem_ under severe water deficit (WS2). Mean values for the different rootstock genotypes studied are represented by different colours, with triangles indicating rootstocks studied in 2021 and squares representing those studied in 2022. The legend includes the linear regression lines for each year and the combined data from both seasons. *, ** and ** mean p-value of <0.05, <0.01, and <0.001, respectively.

Seeking links between root morphological traits and water relations, regressions were explored between the studied traits and Ψ_stem_ for each water stage in both years (**Supplementary Figure 2**). The most significant relationships were found between the percentage of RLD generated by fine roots (ø< 0.5 mm) and Ψ_stem_ under severe water deficit (WS2) conditions (**Figure 6**). Nonetheless, the strength of these linear regressions was low, but positive and with similar slope in both the genotypes studied during 2021 and 2022. Furthermore, when examining this relationship including both seasons together, the positive correlation between the variables increased to moderate (r^2^ = 0.46), showing a higher slope than that of separate seasons.

**Figure 7:**
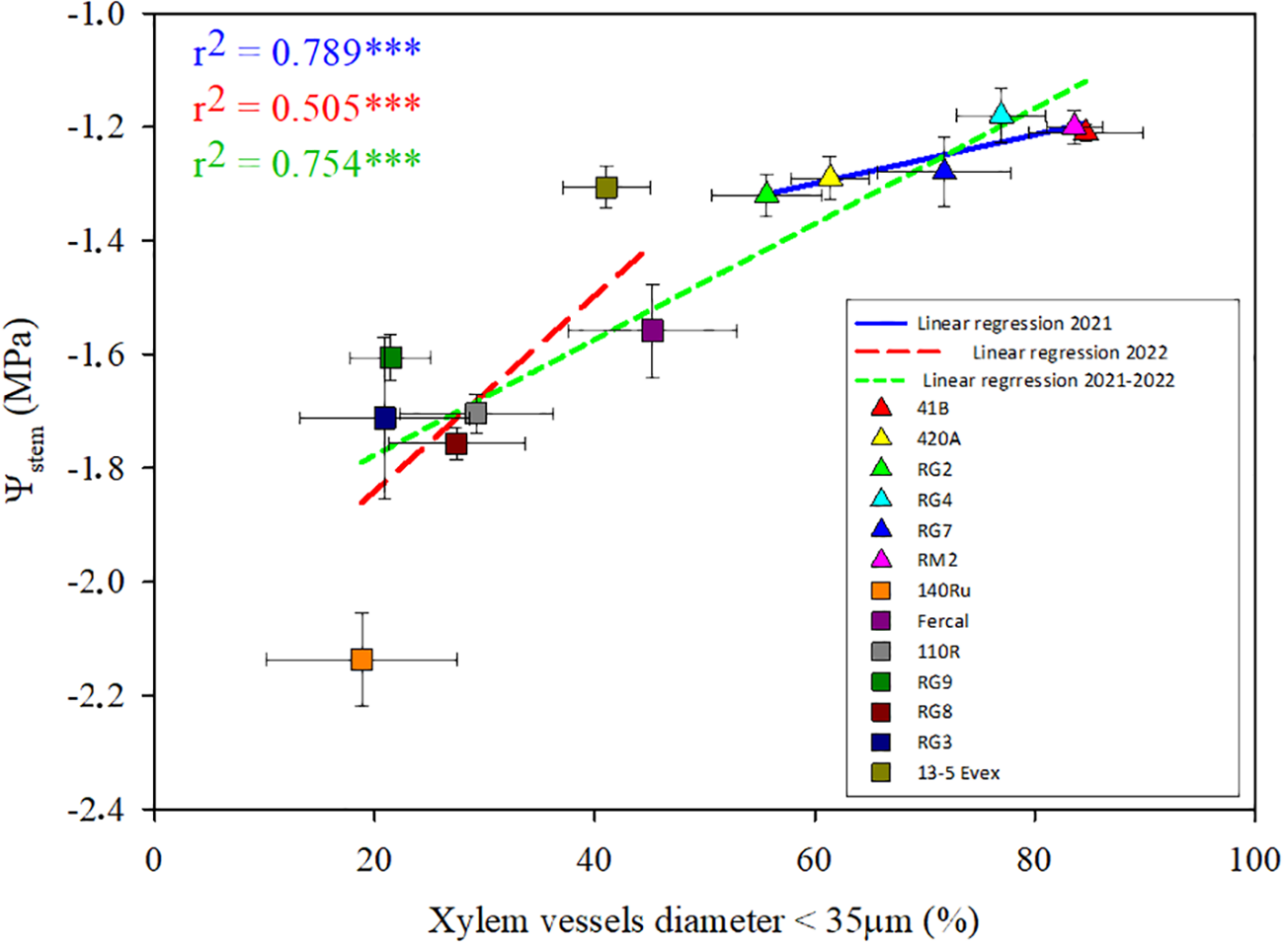
The figure depicts a multiple linear regression analysis of the percentage of xylem vessels with a diameter thinner than 35 μm against Ψ_stem_ under severe water deficit. Mean values for the rootstocks studied are differentiated by colours, with triangles representing rootstocks studied in 2021 and squares representing those studied in 2022. The linear regression lines for each year and the combined data from both years are illustrated in the legend. *** means p-value <0.001.

Similarly, seeking links between root anatomical traits and water relations, regressions were also explored between the studied traits and Ψ_stem_ for each water stage (**Supplementary Figure 2**). The most significant relationships were found between the percentage of fine xylem vessels (D< 35 µm) and Ψ_stem_ under severe water deficit (WS2) (**Figure 7**). These positive correlations were strong in both season data sets (r^2^ > 0.5). When plotting together the rootstocks studied in 2021 and 2022, the strength of the relationship did not improve, showing an average slope between both years.

## 4 Discussion

This work sought to evaluate how root system morphology and anatomy affects the regulation of plant water status, focusing on grapevine rootstocks. Given the complexity and environmental responsiveness of root, experiments were conducted on plants with newly formed roots to control water conditions during root formation (Bauerle et al., 2008; Amulya et al., 2024). Regarding our experimental design, the trial was carried out in own-rooted rootstocks to isolate the genotype effect on hydraulic regulation of vine transpiration, independent of scion interaction (Zhang et al., 2016). Conducted in pots with organic substrate, the experimental design standardized soil volume, water availability and soil properties, facilitating the study of soil water deficit effects on root traits (Passioura, 2006). The time of root sampling for morpho-anatomical determinations was after the recovery period, so as not to affect the water recovery process of the plant. However, new roots might have been generated during the recovery period. Nevertheless, the effect of root growth during this short period is considered to be negligible, as for anatomical analysis, only lignified roots were sampled at 8-10 cm from the root tip; and for the morphological analysis, only the lignified roots were identified by the scanner after extraction and washing. The experiment was conducted in two years, with the same layout but different genotypes, including well-known commercial rootstocks: 420A, 41B, 13-5 Evex, 140Ru, 110R, and Fercal (Serra et al., 2014; Ollat et al., 2015; Marín et al., 2021); and newly bred rootstocks, hybrids of 41B × 110R (RG2, RG2, RG4, RG6, RG7, RG8, and RG9) and RM2 (Ramsing et al., 2021; Buesa et al., 2023; Marín et al., 2023). Despite the experiment did not aim to compare seasons, in most of the physiological, morphological and anatomical variables studied there was a clear year effect. Consequently, inter-annual differences have also been discussed.

### 4.1 Rootstock physiology to soil water deficit and recovery

As plant water status indicator, stem water potential (Ψ_stem_) was chosen for its widespread use at commercial level and its reliability as a crop water status reference (Choné et al., 2001; Levin, 2019). As expected, results from both years showed that with the progressive imposition of water deficit in the soil, Ψ_stem_ transitioned from less negative values under well-watered (WW) conditions to more negative values under severe water stress (**Figure 3**). Water potential measurements at WW, moderate water deficit (WS1) and severe water deficit (WS2) stages were taken after maintaining the same daily irrigation schedule for at least a fortnight, ensuring that genotypes were under similar soil water conditions. The attained levels of water potential indicate that the vines transited from non-stressed to severe water stress in both seasons (Romero et al., 2010). At the WW stage, Ψ_stem_ indicated no water stress in vines across the two years (Cifre et al., 2005). However, the average Ψ_stem_ in 2022 was more negative than those in 2021 at the same water stage, likely due to differences in vine vigor between genotypes (Rogiers et al., 2012) and in climatic conditions between seasons (**Supplementary Figure 3**). Despite these slight differences, all Ψ_stem_ values during WS2 in 2021, were low enough to be considered stressful for this species (Cifre et al., 2005; Romero et al., 2010). Furthermore, upon rehydration at the end of the drought cycle, all genotypes in both years successfully recovered, reaching values close to their initial states (**Figure 3**), suggesting no hydraulic damage and rapid decline in abscisic acid levels upon rewatering (Hochberg et al., 2016; Ruehr et al., 2019; McAdam, 2023).

Regarding the differences in water status among genotypes, in 2021, the Ψ_stem_ was similar under WW and WS1 conditions, but slight differences emerge under WS2 and, remarkably, during recovery (**Figure 3**). The differences among genotypes in 2021 were not fully consistent across the experiment. For instance, at the WS2 stage, RG2 showed the most negative Ψ_stem_, whereas RM2 the least, while during R1 and R2 both genotypes showed intermediate Ψ_stem_ values. In fact, during the recovery stages, the RG4 showed significantly less negative Ψ_stem_ values than 41B in R1 and those of RG7 in R2. In 2022, genotypes did show slight differences from the very first stage (WW), becoming more pronounced as soil water deficit progressed. Remarkably, at the end of the recovery period (R2), no differences between rootstocks were achieved. RG8 tended to show among the most negative Ψ_stem_ values over the season, from WW to R1, but at WS2, 140Ru had the significantly lowest Ψ_stem_, while 13-5 Evex had the least negative. This is in line with Lavoie-Lamoureux et al. (2017) who reported that *V*. *rupestris*-based rootstocks induced lower Ψ_leaf_ compared to *V*. *riparia*-based ones (**Figure 1**). Moreover, Fercal genotype was the one that recover faster its water status, a hallmark of water stress tolerance (Fort et al., 2017). Remarkably, Fercal comes from crosses of *V. Berlandieri* with *V. longii* and *V. vinifiera*, respectively (**Figure 1**).

Previous studies suggest that grapevine rootstock genotypes handle water shortage differently due to variations in root morphology (Peccoux et al., 2017; Pérez-Álvarez et al., 2023; Amulya et al., 2024). In order to identify which root traits influence water uptake we examined the most significant anatomical and morphological traits, focusing on young roots (Zhu et al., 2017; Cuneo et al., 2021).

### 4.2 Root traits: morphological and anatomical diversity for drought stress tolerance

As expected, a remarkable diversity in root morphology and anatomy among genotypes was found in both years (**Tables 1**, **2**) (Mapfumo et al., 1994; Ramsing et al., 2021; Fanton et al., 2024). Total root length, root dry mass and root length density (RLD) were larger in 2021 than in 2022, whereas, xylem diameter, theoretical specific conductivity (*k*_th_), and A_xyl_-to-A_tot_ ratio were smaller. These differences may have been influenced by differences in the environmental conditions between seasons and by differences in the vine water status levels imposed and its duration. Namely, higher vine water stress reached in 2022 than in 2021, but also more demanding conditions (i.e. ET_o_) (**Supplementary Figure 3**).

Regarding root morphology, the results revealed significant differences among genotypes (**Table 1**). In 2021, genotype 420A displayed the most extensive root system, with high abundance of fine, densely packed roots, leading to the highest total root length, dry mass, and density. However, these differences did not result in a more favorable water potential (Ψ_stem_) compared to other genotypes (**Figure 3**). Conversely, RG7, with the least developed root system, did exhibit the poorest recovery of Ψ_stem_ after drought. This aligns with the established role of fine roots in water uptake in grapevine rootstocks (Zhang et al., 2016; Cuneo et al., 2021). Supporting this, in 2021, under WS2, the RG2 genotype suffered the higher water stress and showed the lowest percentage of fine roots, whereas RM2, experiencing significantly milder stress, showed the highest percentage of fine roots (**Figure 4**). Similar trends were observed in 2022; with Fercal showing a significantly higher total root length, dry mass, RLD and SRA, displayed the fastest recovery (**Figure 3**). On the contrary, genotype 110R, with lower root development, did not show more negative Ψ_stem_. In this sense, less dense root systems are expected to be less able to explore water from the soil, so they would have to reach more negative Ψ_stem_ to be able to extract water in the soil regions they explore (Brillante et al., 2016). However, the 110R is well-known to be a drought tolerant rootstock (Pou et al., 2008; Tramontini et al., 2013; Ollat et al., 2015; Peccoux et al., 2017) but in line with our results, it has been characterized by large root diameters low root development under deficit irrigation conditions in pots (Reingwirtz et al., 2021; Pérez-Álvarez et al., 2023). There are other morpho-anatomical traits not studied in this work that also appear to drive root water uptake, such as the proportion of white-functional roots and lacuna formation under water deficit (Cuneo et al., 2021; Reingwirtz et al., 2021).

Analysis of root anatomy also revealed variations among the studied genotypes. In 2021, genotype 420A was found to have the highest xylem diameter, *k*_th_, and A_xyl_-to-A_tot_ ratio (**Table 2**). On the other hand, genotype 41B exhibited the lowest values for the main xylem anatomical traits. However, both genotypes did not differ on its Ψ_stem_ compared to the other genotypes (**Figure 3**). In 2022, RG8 and Fercal displayed the highest and lowest percentages of A_xyl_-to-A_tot_, respectively (**Table 2**). These genotypes also exhibited the slowest and fastest recovery from water stress at R1 stage, respectively. However, RG8’s difficulty in recovering the initial water potentials might be due to suberin deposits in the root epidermal cells forming an impermeable barrier, decreasing hydraulic conductivity (Zhang et al., 2016; Cuneo et al., 2021), resulting in differences between grapevine rootstocks (Barrios-Masias et al., 2015). Moreover, in 2022, the genotypes with the lowest and highest xylem V_D_, 140Ru and 13-5 Evex, respectively, reached the most and less negative Ψ_stem_ values at WS2. This is supported by the well-known relationship between higher V_D_ and hydraulic efficiency (Waite et al., 2023). However, it was also found that in the distribution of xylem vessel sizes, the 140Ru showed significantly lower percentage of small vessels (<35 μm) than 13-5 Evex (**Figure 5**), which in turn, would be associated with a higher vulnerability to embolism of 140Ru compared to 13-5 Evex (Anfodillo and Olson, 2021; Isasa et al., 2023).

Common patterns in plant water relations and root morphological and anatomical traits across genotypes were found. For instance, total root length was overall positively related to total root dry mass and RLD (**Supplementary Figure 2**). That is, genotypes with higher percentage of fine roots (D< 0.5 mm) (**Figure 4**) also generate more root biomass (**Table 1**). Certainly, the maintenance of root growth during water deficit stages, could explain differences among genotypes on root biomass, is considered a trait of tolerance to water stress (Bauerle et al., 2008; Fort et al., 2017). Remarkably, there was an overall positive correlation between RLD and Ψ_stem_ across water stages, particularly at WS2 when considering only the fine roots (**Figure 6**). Furthermore, the opposite is true for xylem diameter and Ψ_stem_, with higher xylem diameters related to more severe stress of the genotype under severe water deficit and recovery (**Supplementary Figure 2** **;** r^2^ = -0.53, -0.66 and -0.52 at WS2, R1 and R2, respectively). Moreover, irrespective of the seasonal effect on xylem diameter, an elevated percentage of root xylem vessels measuring less than 35 µm was related to lower water stress across genotypes (**Figure 7**). These was also observed in a commercial vineyard of ‘Merlot’ onto 140Ru by Munitz et al. (2018). These authors reported that higher irrigation rates resulted in wider xylem vessels in the stems and greater sensitivity to late season water stress (i.e. more negative Ψ_stem_). In fact, in *Vitis vinifera* and other tree species, these reductions in xylem vessel size have been observed at the stem level as a drought of acclimation mechanism and have been termed “water stress structural memory” (Tombesi et al., 2018; Netzer et al., 2019). However, during this acclimatation process, Shtein et al. (2021) indicated a positive relationship between xylem structure of petioles of *Vitis vinifera* L. ‘Cabernet Sauvignon’ and stomatal conductance (g_s_), but not with Ψ_stem_. They related this to a reduction of xylem vessels >30 μm by temperature effect, which was related to g_s_ but not to Ψ_stem_.

These findings suggest a trade-off where higher root density (i.e. RLD; **Figure 6**) enhances water uptake capacity but with penalties on water transport capacity (i.e. *k*_th_; **Figure 7**). Consequently, average root diameter, inversely related to RLD (**Supplementary Figure 2**; r^2^ = 0.60), also plays a crucial role in this process (Mapfumo et al., 1994; Gambetta et al., 2013; Pouzoulet et al., 2014). It is well-known that increased RLD provides more root-soil contact, facilitating water uptake (de Herralde et al., 2006; Cuneo et al., 2021), but roots with small diameters may struggle to efficiently transport the absorbed water (Tyree and Ewers, 1991; Brunner et al., 2015). This is supported by the positive relationships between root diameter and A_xyl_-to-A_tot_ ratio (**Supplementary Figure 2**; r^2^ = 0.40), and the latter with *k_th_* (**Supplementary Figure 2**; r^2^ = 0.75). In this sense, low A_xyl_-to-A_tot_ ratio, which was found to be related to low root diameter, induced faster recovery from severe water deficit (i.e. Fercal and RG4). The importance of fine roots lies in their high surface area-to-volume ratio, making them highly efficient structures for water uptake (Gambetta et al., 2013). In essence, RLD is a significant factor affecting plant response to water deficit due to its influence on water uptake, but it needs to be balanced with proper root diameter for optimal water transport throughout the plant.

Therefore, both morphology and anatomy underlined significant relationships with plant water status across grapevine rootstock genotypes. However, the relationship between root morphology and Ψ_stem_ was mild, which might be explained because root density plays a reduced role in a small soil volume of the pots compared to the field. In any case, the morpho-anatomical relationships with vine water status became clearer under severe water stress conditions (WS2). The weaker relationships under higher soil water availabilities could be attributed to the complexity of the mechanisms deployed by plants to regulate their water status, with different anatomical, mechanical, and chemical processes shaping plant hydraulics (Chaves et al., 2010; Gambetta et al., 2020). In fact, under less water-limiting soil conditions, other physiological processes seem to play a more prominent role, i.e. stomatal and mesophyll conductances (Escalona et al., 2000; Flexas et al., 2002). For this reason, the relationship between Ψ_stem_ and g_s_ shows only a moderately strong correlation (**Supplementary Figure 4**). Under mild water stress, stomatal control of transpiration may buffer the effect of morpho-anatomy *per se*, since it is only when stomata are practically closed that they become evident. That said, stomatal regulation in commercial vineyards depends on the scion, i.e. *Vitis vinifera* L., rather than on the rootstock genotypes evaluated in this study. Nevertheless, rootstocks are recognized to mediate regulation via hormonal and/or osmotic pathways (Ollat et al., 2015; Zhang et al., 2016; Marín et al., 2021). Therefore, the fact that when stomatal control loses importance in water relations it is root morpho-anatomical traits that govern vine water status suggests that the influence of these traits is likely to occur similarly in commercial vineyards since they usually face severe drought conditions (van Leeuwen et al., 2024).

Previous studies have also found significant relationships between hydraulic traits (e.g., hydraulic conductivity and xylem embolism vulnerability) and anatomical traits in grapevine genotypes only under specific conditions (Lovisolo and Schubert, 1998; Venturas et al., 2016; Pouzoulet et al., 2019; 2020). For instance, Lamarque et al. (2023) found that only the 50% loss of hydraulic conductivity due to very severe water stress correlated with *k_th_*, with grapevine cultivars and rootstocks exhibiting lower vulnerability to xylem embolism showing lower *k_th_*. In line with this, Isasa et al. (2023) reported that the embolism resistance (as measured by P_50_) across 77 trees species was positively related to xylem vessels diameter. Similar trend was recently observed in conifer embolism resistance from the stem apex to base (Zambonini et al., 2024). In our experiment, smaller xylem vessels, also related to lower *k_th_*, prevent from reaching very negative Ψ_stem_, thereby avoiding reaching water potential thresholds susceptible to induce cavitation (Dayer et al., 2020a). Indeed, small vessels are theoretically less susceptible to the formation of embolism events caused by water scarcity (Pouzoulet et al., 2020; Quintana-Pulido et al., 2018). This was recently corroborated by Sorek et al. (2021), reporting a reduced vulnerability to embolism as leaf xylem size decreased. However, Pagay et al. (2016) reported that shoots with larger xylem diameter vessels were less vulnerable to xylem cavitation than shoots with smaller vessels, attributed to lower xylem inter-vessel pitting in shoots with larger xylem diameter vessels (Kaack et al., 2021; Isasa et al., 2023). Pit membranes are indeed correlated with trade-offs between hydraulic efficiency and resistance (Choat et al., 2008; Pouzoulet et al., 2014). Specifically, related to the number of pit membranes, as well as their diameter-to-thickness ratio (Levionnois et al., 2021) and irregularities in pit structure (Plavcová et al., 2013).

Therefore, our results with grapevine rootstocks provide mechanistic evidence for the existence of implications of effects of root morphology and anatomy on water deficit tolerance and recovery. These findings focus on tolerance to water deficit rather than specifically addressing resistance to xylem cavitation. However, given the levels of water deficit reached in our experiment, it is hypothesized that rootstocks capable of avoiding embolism will be those that recover first in terms of water status (McAdam, 2023; Flor et al., 2025). Future work focusing on the analysis of root morpho-anatomical traits involved in the regulation of water status should identify the functional traits associated with both water deficit tolerance and resistance (Hochberg et al., 2016; Villalobos-Soublett et al., 2022).

## 5 Conclusions

The findings of this study underscore that the intricate nature of water deficit tolerance and recovery responses is genotype-dependent. A trade-off between root architecture and anatomy effects on vine water status is particularly noteworthy. Root length density (i.e. the proportion of fine roots) and xylem vessel diameter regulate water uptake from the soil and water transport through the plant, respectively. Across the 13 genotypes evaluated, a higher root length density (RLD) was slightly related to milder Ψ_stem_, while a higher proportion of smaller xylem vessels was related to less negative potentials. This phenomenon is particularly evident under severe water deficit conditions, when stomatal regulation plays little role in relation to hydraulic traits, but also during the period of water recovery following such conditions. It can be concluded that 420A, 41B, RM2 and Fercal genotypes appear to be promising grapevine rootstock candidates, while also providing new insights for future breeding programs. The new RG-rootstocks did not display any particularly interesting traits for adaptation to water stress compared to commercial rootstocks. However, further research is required in order to confirm these results and expand knowledge on grapevine drought stress responses under non-limiting soil conditions in the field and under grafted conditions.

## Data Availability

The raw data supporting the conclusions of this article will be made available by the authors, without undue reservation.

## Supplementary material

Supplementary Figure 1 Cross-section of a root of RG4 grapevine rootstock.

Supplementary Figure 2 Correlation matrix between root anatomy and morphology traits and Ψ_stem_ at each water stage averaged across the genotypes. Data are the Pearson coefficient of the linear regression between variables. *, **, and *** mean statistically significant relationship at p<0.05, p<0.01, and p<0.001, respectively. The distribution of the data for each variable is depicted in the center of the graph.

Supplementary Figure 3 Meteorological variables over the experimental seasons of 2021 **(A)** and 2022 **(B)** in the experimental site (Palma, Balearic Islands, Spain). Daily mean temperature (red) and reference evapotranspiration (blue).

Supplementary Figure 4 Linearized relationship between stomatal conductance (g_s_) and stem water potential (Ψ_stem_) using the natural logarithm of the g_s_ data measured at leaf basis on the 13 genotypes in the two experimental seasons (●, 2021 and ▼, 2022) under pot conditions in Palma, Balearic Islands, Spain. Fitted regressions for each year are shown as well as the Pearson coefficient for each of them. Asterisks indicate significance of the regression (P< 0.0001).
