# Supplementary figures and images for: Implications of root morphology and anatomy for water deficit tolerance and recovery of grapevine rootstocks
Source: Front Plant Sci. 2025 Mar 20;16:1541523. doi: 10.3389/fpls.2025.1541523 (PMC11966617; doi:10.3389/fpls.2025.1541523)

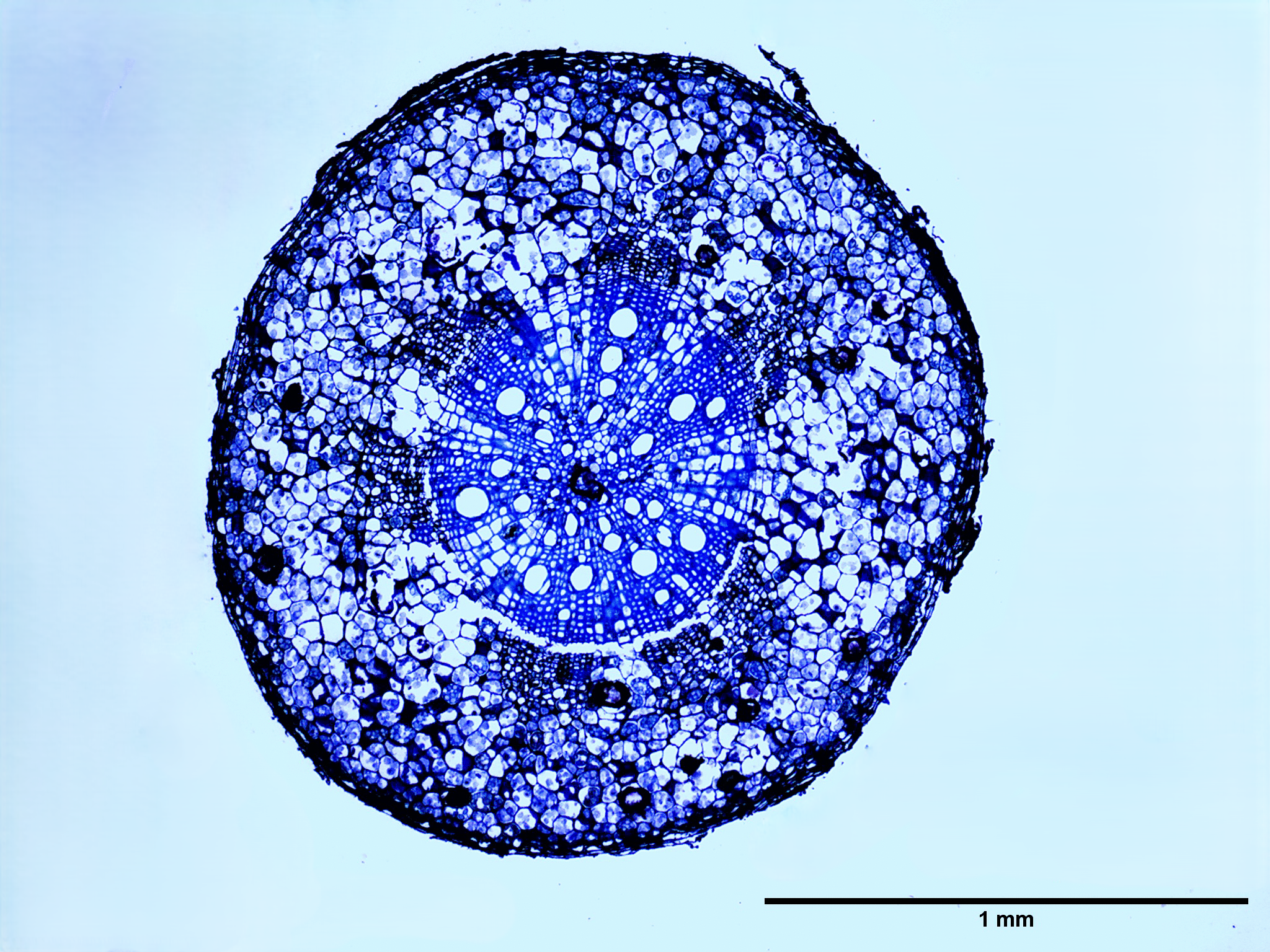

Supplement: Supplementary Figure 1 — Cross-section of a root of RG4 grapevine rootstock. [file Image1.tiff]

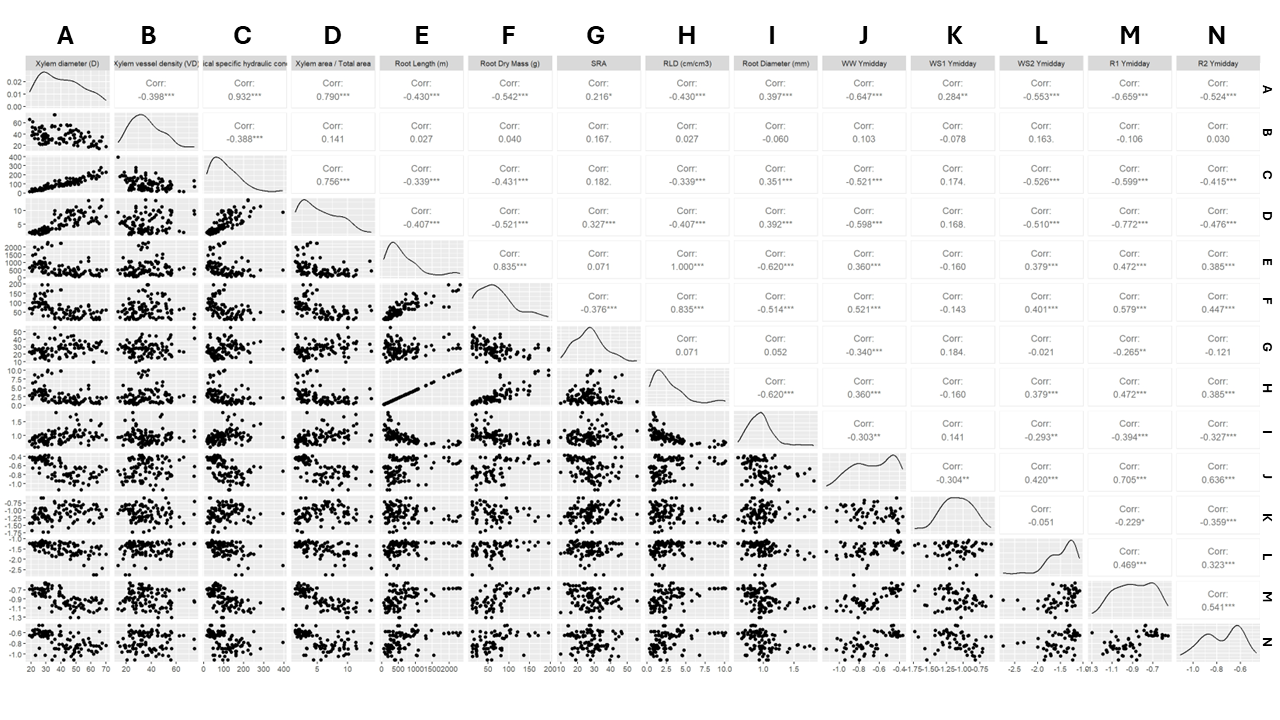

Supplement: Supplementary Figure 2 — Correlation matrix between root anatomy and morphology traits and Ψstem at each water stage averaged across the genotypes. Data are the Pearson coefficient of the linear regression between variables. *, **, and *** mean statistically significant relationship at p<0.05, p<0.01, and p<0.001, respectively. The distribution of the data for each variable is depicted in the center of the graph. [file Image2.tif]

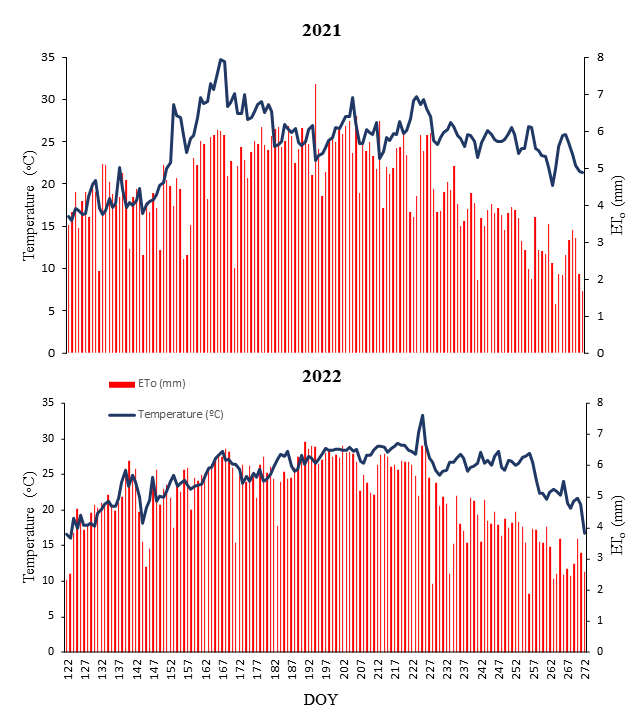

Supplement: Supplementary Figure 3 — Meteorological variables over the experimental seasons of 2021 (A) and 2022 (B) in the experimental site (Palma, Balearic Islands, Spain). Daily mean temperature (red) and reference evapotranspiration (blue). [file Image3.tif]
